# COMPARATIVE TRANSCRIPTOMIC OF LONGEVITY

**DOI:** 10.1093/geroni/igad104.1423

**Published:** 2023-12-21

**Authors:** Vera Gorbunova

## Abstract

Transcriptome analysis provides a nuanced view into the changes that occur in cells and tissues. Transcriptome changes rapidly and reproducibly in response to physiological influences and environmental insults. Recent years have seen an exponential increase in transcriptome data at bulk, single cell and spatial resolution that allows insights into the mechanisms and regulatory pathways of aging and longevity. In this session Drs. Gorbunova (University of Rochester) and Gladyshev (Harvard Medical School) will discuss comparative transcriptomics of longevity across species with diverse lifespans that revealed unique signatures of longevity and the integration of transcriptome and proteome data. Dr. Gladyshev will discuss development of transcriptomic clocks of measuring biological aging. Dr. Artyomov will discuss single-cell resolution approaches to reveal aspects of immune aging in humans, and Dr. Palovics will present the use of transcriptomics to understand rejuvenating effects of heterochronic parabiosis.

